# Uncovering Latent Deficits Due to Mild Traumatic Brain Injury by Using Normobaric Hypoxia Stress

**DOI:** 10.3389/fneur.2013.00041

**Published:** 2013-04-30

**Authors:** Leonard Temme, Joseph Bleiberg, Dennis Reeves, David L. Still, Dan Levinson, Rebecca Browning

**Affiliations:** ^1^Vision Sciences Branch, Sensory Research Division, U.S. Army Aeromedical Research LaboratoryFort Rucker, AL, USA; ^2^National Intrepid Center of Excellence, Walter Reed National Military Medical CenterBethesda, MD, USA; ^3^Clinvest ResearchSpringfield, MO, USA; ^4^California School of Forensic Studies, Alliant International University IrvineIrvine, CA, USA

**Keywords:** mild traumatic brain injury, normobaric hypoxia, cognitive stress test, concussion, BrainCheckers, automated neuropsychological assessment metrics, mTBI biomarkers, hypoxic challenge

## Abstract

Memory deficits and other cognitive symptoms frequently associated with mTBI are commonly thought to resolve within 7–10 days. This generalization is based principally on observations made in individuals who are in the unstressed environmental conditions typical of a clinic and so does not consider the impact of physiologic, environmental, or psychological stress. Normobaric hypoxic stress can be generated with normal mean sea level (MSL) air, which is about 21% oxygen (O_2_) and 78% nitrogen (N), by reducing the percentage of O_2_ and increasing the percentage of N so that the resultant mixed-gas has a partial pressure of O_2_ approximating that of specified altitudes. This technique was used to generate normobaric hypoxic equivalents of 8,000, 12,000, and 14,000 feet above MSL in a group of 36 volunteers with a mTBI history and an equal number of controls matched on the basis of age, gender, tobacco smoking consumption, weight, height, and body mass index. Short-term visual memory was tested using the Matching to Sample (M2S) subtest of the BrainCheckers analog of the Automated Neuropsychological Assessment Metrics. Although there were no significant differences in M2S performance between the two groups of subjects at MSL, with increased altitude, the mTBI group performance was significantly worse than that of the control group. When the subjects were returned to MSL, the difference disappeared. This finding suggests that the “hypoxic challenge” paradigm developed here has potential clinical utility for assessing the effects of mTBI in individuals who appear asymptomatic under normal conditions.

## Introduction

Memory deficits and other cognitive problems are associated with mTBI, which is often alternatively referred to as concussion (Randolph et al., 2009). The literature on the recovery from concussion suggests that the duration of symptoms usually is from 7 to 10 days (McCrory et al., 2005). Almost invariably these conclusions are based on observations made in individuals under unstressed environmental conditions and so do not address the impact of stresses encountered in the operational environment. Consequently, there is limited information regarding how individuals with a history of mTBI function when stressed. This raises the possibility that individuals with an mTBI history may seem completely asymptomatic during standard clinical examination but may become symptomatic when exposed to such stress as sleep deprivation, pharmaceuticals, extreme temperature, anxiety, hypoxia, or other stressors that affect brain function.

The hypothesis that physiologic or psychological stress might uncover latent or subclinical mTBI symptoms was suggested by an incidental observation made in a flight simulator during a study of instrument flight by highly skilled, military instructor pilots who were exposed to a normobaric hypoxic condition that approximated conditions encountered at 18,000 feet above mean sea level (MSL) (Temme et al., 2010). Under the control condition of breathing MSL air, which contains about 21% oxygen (O_2_) and 78% nitrogen (N), the flight performance of all pilots was essentially indistinguishable and consistent with their extraordinary expertise; however, when the pilots were breathing a normobaric simulation of air 18,000 feet above MSL, which contained about 9.98% O_2_ and 89% N, one pilot not only lost control of the aircraft but did not realize that fact. Subsequent review of the pilot’s medical record with the medical monitor who cleared the pilot for the study showed that some years earlier the pilot had experienced a significant concussion while ejecting from a high-performance aircraft.

Support for this hypothesis was found in a study of a group of 10 university students who had experienced “minor head injury” 1–3 years prior to the study (Ewing et al., 1980). At the time of the study, these students were asymptomatic and performing comparably to pre-injury levels. These students, along with a control group of 10 age-, gender-, and academic performance-matched students, were exposed for 30 min to an altitude stress of 3800 m (about 12,500 feet) above MSL in a low-pressure chamber. None of the students in the experimental group was symptomatic at MSL; however, at altitude, there were clear deficits in short-term memory and judgment as reflected by a more lax response bias in a signal detection task. This detection task permitted the differentiation of response bias from sensitivity, which was not affected by the altitude stress. Some effects were apparent within 10 min of altitude exposure. These effects were reversible, disappearing when the students were returned to MSL (Ewing et al., 1980).

The purpose of the present study was to see whether normobaric hypoxic stress had a differential effect on the neurocognitive performance of mTBI and control subjects.

## Materials and Methods

The study was performed at Clinvest Research, Springfield, MO, USA, following review and approval by the Chesapeake Institutional Review Board, the U.S. Army Aeromedical Research Laboratory Human Use Committee, and the U.S. Army Medical Research and Materiel Command Human Subjects Research Review Board. The study was conducted in accordance with all Federal laws, regulations, and standards of practice as well as those of the Department of Defense and U.S. Army. The study was determined to pose a greater than minimal risk to the subjects and included several risk mitigation techniques. One such technique was to present the normobaric hypoxic stress conditions in an ascending sequence of severity so that each subject was observed at a lower stress condition before being exposed to a greater stress.

### Subjects

Subject recruitment flyers were posted throughout the local community where people with a history of mTBI would likely see them. These locations included medical as well as sports facilities such as athletic and health clubs and gymnasiums. The flyers briefly described the study and included a contact phone number. During initial phone screening, the study and the criteria for participation were further described, an appointment was scheduled, and the amount of financial compensation for study participation stated. Prospective subjects were told to abstain from any alcohol consumption for at least 12 h prior to testing. A copy of the Informed Consent Document was mailed or e-mailed to the prospective subject to review before arriving at the research center for the study.

Subjects consisted of two groups of 36 subjects each, one group with a history of mTBI and one without. Subjects were between the ages of 18 and 50 years. Specific exclusion criteria were pregnancy; history of drug or alcohol abuse; depression; bipolar disorder; schizophrenia; heart, kidney, or liver disorders; asthma; strokes or mini-strokes; poor leg circulation; current or past neurological problems such as seizures, epilepsy, dementia, or post-traumatic headache; current concentration and/or memory problems secondary to the mTBI; loss of consciousness longer than 10 min at the time of injury; and post-traumatic amnesia greater than 24 h. While medical records and other source documentation were requested, these were not available in most cases and much of the information was by subject self-report, a method with known weaknesses (Rapp and Curley, 2012).

The median interval between the trauma of an mTBI subject and that subject’s testing was 1.9 years (mean = 3.1; SD = 2.7 years), with the first and the third quartile 1.3 and 4.7 years respectively. The smallest interval was 0.6 years and the largest interval was 9.7 years. Of the 36 mTBI subjects, 15 reported a loss of consciousness averaging about 4 min in duration and ranging between a few seconds to a few minutes. Five subjects reported some form of post-traumatic amnesia with a duration ranging from about 5 min to a couple of hours. Sports accidents accounted for 11 traumas, motor vehicle accidents accounted for 10 traumas, falls accounted for 5 traumas, head strike accounted for 3 traumas, blast of an improvised explosive device experienced in Iraq accounted for 1 trauma, and 6 traumas were not described in the records. The one subject with the history of blast mTBI, was receiving treatment for post-traumatic stress disorder (PTSD); no other subjects reported PTSD and no subject reported multiple TBIs.

The mTBI and control subjects were explicitly matched on the basis of age, gender, tobacco smoking consumption, weight, height, and body mass index, resulting in two groups, each with 9 women and 27 men. No subjects and no data were excluded, so complete datasets were obtained and analyzed from a total of 36 volunteers in each of the two groups. Effectiveness of matching is shown in Table 1, which presents group comparisons across seven parameters. Multivariate analysis failed to show any statistically significant differences between the groups on any of the parameters presented in Table 1. Further, pairwise probabilities corrected for multiple comparisons with the Bonferroni method ranged between 0.180 and 0.817.

**Table 1:** **Comparison of physical parameters between groups**.

		Age (years)	Pulse (bpm)	Blood pressure (mmHg)		Weight (kg)	Height (m)	Body mass index
				Systolic	Diastolic			
mTBI	Mean	25.3	70.8	120.1	73.9	90.8	1.8	28.3
	SD	5.4	11.6	14.8	9.8	29.4	0.1	7.9
Control	Mean	24.9	72.7	120.9	73.4	95.7	1.8	29.7
	SD	4.9	14.2	14.6	7.8	38.8	0.1	11.5

In order to minimize the impact of possible subtle methodological shifts over time due to extraneous factors such as changes in staff, experimenter expertise or bias, procedures, criteria, or instrumentation drift, each mTBI subject was tested as close in time as possible to the matched control subject. The median interval between testing of the mTBI subject and the matched control subject was 4 days, with the interval between the first quartile and the third quartile ranging from 1 to 8 days, and the maximal interval being 25 days.

### Instrumentation

#### Reduced oxygen breathing device-2

The reduced oxygen breathing device (ROBD)-2 is a commercially available, off-the-shelf device that simulates altitude by using normobaric hypoxia. The normobaric hypoxia is generated from MSL air, which is normally about 21% oxygen (O_2_) and 78% nitrogen (N), by reducing the percentage of O_2_ and increasing the percentage of N so that the resultant mixed-gas has a partial pressure of O_2_ approximating that of specified altitudes (Still and Temme, 2012). This method permits the generation of altitude-related hypoxic states without using a low-pressure chamber; and therefore without exposing an individual to changes in barometric pressure and its associated risk of decompression sickness. The target altitudes used in the present study were 8,000, 12,000, and 14,000 feet above MSL, with % O_2_ of about 15.5%, 13.0, and 12.0%, respectively, in addition to the 21.0% O_2_ typical of MSL. The complete technical description, including blueprints and engineering specifications of the ROBD-2 is available from the manufacturer’s website (Environics, 2012).

#### Neuropsychological evaluation

Cognitive performance was assessed with selected neuropsychological subtests of BrainCheckers, the Automated neuropsychological assessment metrics (ANAM) readiness evaluation system (ARES), which is a Palm OS analog of ANAM (Elsmore et al., 2007; Reeves et al., 2007). These subtests were administered on a Palm Tungsten 2E handheld Personal Digital Assistant (PDA). The present study used eight tests from the BrainCheckers test battery: (1) Sleep Scale, (2) Simple Reaction Time, (3) Procedural Reaction Time, (4) Running Memory, (5) Matching to Sample (M2S), (6) Congruent Stroop, (7) Incongruent Stroop, and (8) Pursuit Tracking. For all subjects at each altitude the BrainCheckers tests were administered consistently in the sequence listed above.

(1) Sleep Scale: This test is a simple self-rating of the subject’s current feeling of alertness rated on a seven-step scale. The subject is instructed to read all seven statements and choose the one that applies most accurately at the moment. The Sleep Scale reports the numerical rating that reflects alertness.
(2) Simple Reaction Time: An asterisk-like symbol appears on the display screen at varying (650–1350 ms) time intervals. The subject taps the asterisk with a stylus as quickly as possible upon seeing the stimulus. There are 4 practice trials followed by 20 test trials. The Simple Reaction Time reports throughput, which is the number of correct responses per minute.
(3) Procedural Reaction Time: This is a measure of choice reaction time that requires the subject to differentiate between two sets of characters. The test presents a numeric stimulus on the screen, which is a 2, 3, 4, or 5. The subject’s response is a tap on one of two buttons; one button to indicate that the stimulus was a 2 or 3 while the other button indicates that the stimulus was a 4 or a 5. The test is preceded by 4 “warm-up/practice” sessions; 32 stimuli are presented during the test. The Procedural Reaction Time reports throughput, the number of correct responses per minute.
(4) Running Memory Continuous Performance test: This task requires the subject to indicate whether the current number is the number that had just been displayed. The “SAME” button is to be pressed if they are the same and the “DIFF” button is to be pressed if the number is different. The numbers match on 50% of the trials and a number will never be presented on more than three consecutive trials. No response is required to the first stimulus since there is no “preceding” stimulus with which to compare it. Ten warm-up/practice trials are followed by 40 stimuli that comprise the test. To ensure the subject understands this test, the subject must correctly answer 70% of the warm-up/practice trials before the test begins. The Running Memory Continuous Performance test reports throughput, the number of correct responses per minute.
(5) Matching to Sample: This is a test of short-term memory, attention, and visual-spatial discrimination. The subject is presented with a single design for 3 s to study and remember. The design then disappears and the screen goes blank. Following a delay of 5 s, two designs simultaneously appear on the screen. The subject indicates which of the two designs matches the original. This subtest is preceded by two practice stimuli that demonstrate to the subject the audio feedback that is associated with an incorrect response (beep if wrong). The M2S test reports throughput, the number of correct responses per minute.
(6) Congruent Stroop: During this test the words Red, Green, and Blue are presented on the screen in the same color as the word; for example, a red font is used for the word “Red.” At the bottom of the screen are three boxes, one box is labeled R, one G, and one B. The subject taps the box to identify the color of the word. Three warm-up/practice stimuli are followed by 30 stimuli that comprise the actual test. The Stroop test reports throughput, which is the number of correct responses per minute.
(7) Incongruent Stroop: During this subset, the words Red, Green, and Blue are flashed on the screen but in a color different from the word. For example, the word Blue would be presented on the screen using red letters or green letters. The subject taps the response box to indicate the color of the letters of the word. These instructions are identical with the instructions used for the Congruent Stroop but the Incongruent Stroop is harder because the words are in incongruent colors. This test presents 3 warm-up/practice stimuli, followed by 30 stimuli that comprise the actual test. As in the Congruent Stroop above, the Incongruent Stroop reports throughput, which is the number of correct responses per minute.
(8) Pursuit Tracking: This test is a Pursuit Tracking and fine psychomotor abilities assessment. It is designed to detect impairment in fine motor abilities that are evident in Parkinson’s disease and other similar conditions which include intentional tremor, familial tremor, side effects from medication, and cerebellar dysfunction. The test requires pressing the stylus on the screen within a target/bulls-eye and keeping the stylus on the center of the bulls-eye as it moves across the screen in a sine wave pattern for 60 s. The Pursuit Tracking test reports the percent of the time during which the response stylus is on the target.

### Procedures

After the Informed Consent Document was reviewed and signed, a study intake form, which documented past and current medical history, was completed. Female subjects provided a urine sample for pregnancy testing. A physician reviewed the medical history forms and examined the subject to ensure compliance with inclusion/exclusion criteria. The subject was then introduced to the testing facility, including the test apparatus. The technician also fitted the ROBD-2 breathing mask on the subject’s face so that the subject could gain experience using it. When the subject was comfortable breathing MSL air through the ROBD-2 and the mask, the subject was introduced to the BrainCheckers tests. After the subject reported being comfortable with all the testing procedures, s/he was encouraged to take a break before formal testing began.

All subjects went through the same sequence of five simulated altitudes: (1) mean sea level 1 (MSL1), (2) 8,000 feet (2,438 m) above MSL (8k), (3) 12,000 feet (3,657 m) above MSL (12k), (4) 14,000 feet (4,267 m) above MSL (14k), and (5) a second MSL condition (MSL2). As previously noted, the sequence of increasing stress exposures was used as a risk mitigation strategy; however, such a sequence confounds order effects such as training, experience, boredom, fatigue, anxiety, apprehension, and so forth, with the sequence of increasing normobaric stress. This confounding made it impossible to evaluate the effects of one altitude, for example 12k condition, independently of the previous altitude exposures as well as independently of order effects. Thus, the study was not intended to provide an evaluation of the effects of each stress condition on subject cognition. That question presupposes assurance that the methodology could be used safely in such a study. Rather, the present study was intended to assess the feasibility of exposing mTBI subjects to the normobaric hypoxic stress, thereby laying the foundations for future studies.

The MSL1 condition provided the baseline values. The 8, 12, and 14k exposures comprised the hypoxic condition. The MSL2 condition was intended to ensure that whatever effects the hypoxic exposures had were reversible and temporary; MSL2 also served as a safety precaution by providing the opportunity to observe the subject under controlled conditions post-experiment before release.

At each altitude, the subject acclimated for 3 min before beginning the BrainCheckers testing. When testing was completed at each altitude, the subject was returned to MSL and given the option of taking a brief break. If the subject opted for the break, the mask was removed. When the subject was ready to continue, the subject donned the mask, which was checked to ensure a good seal. If the subject opted not to take a break, the subject rested while breathing MSL air through the mask for at least 1 min before proceeding to the next altitude.

### Experimental design and statistical analysis

The goal of the present study was to compare the cognitive performance of the mTBI subjects with that of the control subjects when both groups were exposed to the same normobaric hypoxic stress. That all subjects were exposed to the same sequence of normobaric hypoxia conditions helped ensure that all subjects were exposed equally to the independent variable of interest. The major concern was to ensure that the two groups of subjects were exposed equally to the same hypoxic stress. The question is whether the self-paced nature of the experimental procedures, particularly those that permitted a subject to take a break if desired, compromised this equivalence.

A mixed-model analysis of variance (ANOVA) assessed whether there was any evidence for statistically significant differences between the mTBI and control subjects in the time required for completing the BrainCheckers testing. The means and SDs of the duration of testing in minutes at each altitude for the two groups of subjects are presented in Table 2.

**Table 2:** **Mean (SD) duration in minutes of the BrainCheckers testing for each altitude**.

Subject group	Altitude condition				
	MSL1	8k	12k	14k	MSL2
mTBI	11.28 (1.085)	11.17 (0.737)	11.31 (0.786)	11.11 (0.747)	11.11 (0.820)
Control	11.00 (1.014)	10.97 (0.609)	11.11 (0.887)	10.94 (0.754)	11.14 (0.723)

There was no evidence for a difference between groups [SS Group = 2.336, SS Error = 141.994, *F*(1, 70) = 1.152, *p* < 0.287], nor for a group by altitude interaction [SS Interaction = 0.928, SS Error = 97.7, df = 3.005, 210.369 (Greenhouse–Geisser corrected) *F* = 0.665, *p* < 0.617]. It may also be noted that there was no evidence of any differences among the altitudes.

Although subjects were given the option to take a break after testing at each altitude, the subjects, of their own accord, kept the vast majority of these breaks brief. The means (SD) of the breaks before each of the altitude testing conditions for the two groups are presented in Table 3.

**Table 3:** **Mean (SD) duration in minutes between experimental conditions**.

Subject group	Altitude condition			
	MSL1–8k	8–12k	12–14k	14k–MSL2
mTBI	2.33 (1.00)	3.36 (1.90)	4.78 (3.80)	3.36 (1.90)
Control	2.28 (1.19)	3.44 (1.65)	3.89 (2.80)	3.44 (1.65)

A mixed-model ANOVA showed that there was no evidence for statistically significant differences between the mTBI and control subjects for the intervals in Table 3 [SS Group = 4.253, SS Error = 623.743, df = 1.70, *F* = 0.477, *p* < 0.492], nor for a group by altitude interaction [SS Interaction = 10.372, SS Error = 798.174, df = 2.250, 157.520 (Greenhouse–Geisser corrected) *F* = 0.910, *p* < 0.415]. It may be noted, however, that the duration of the average break preceding 8k condition (2.306 min) was significantly shorter than the breaks preceding 12, 14k, and MSL2 conditions, none of which were different among themselves.

The interval between starting the exposure to the altitude condition and starting BrainCheckers testing was monitored. The means (SD) of these intervals for the two groups at each altitude are presented in Table 4.

**Table 4:** **Mean (SD) duration in minutes between starting the exposure to the altitude condition and starting BrainCheckers testing**.

Subject group	Altitude condition			
	8k	12k	14k	MSL2
mTBI	3.92 (0.6)	4.03 (0.6)	4.14 (0.9)	4.17 (0.9)
Control	4.11 (0.7)	3.97 (0.7)	4.19 (0.9)	4.00 (0.5)

A mixed-model ANOVA showed that there was no evidence for statistically significant differences between the mTBI and control subjects for the intervals in Table 4 (SS Group = 0.003, SS Error = 67.493, df = 1.70, *F* = 0.004, *p* < 0.952) nor for a group by altitude interaction [SS Interaction = 1.288, SS Error = 81.701, df = 2.698, 188.885 (Greenhouse–Geisser corrected) *F* = 1.104, *p* < 0.349].

The above comparisons and analyses of the hypoxic exposure temporal parameters support the conclusion that the temporal parameters of the hypoxic exposure were not statistically different between the two groups.

All data manipulations were performed with Excel 2007 and statistical calculations were performed with SPSS Version 19.

## Results

### Mean sea level 1 baseline

Mean sea level 1 performance provides the baseline for calculating the difference scores used to analyze the effects of normobaric hypoxia on the eight BrainCheckers tests. Specifically, for each test at each altitude condition, the measurement recorded for a specific subject was subtracted from that subject’s performance at MSL1. This technique presupposes that there are no statistically significant differences between the two groups of subjects for any of the tests at MSL1. This precondition was evaluated using a multivariate ANOVA to compare MSL1 performance between the mTBI and control subject for each of the 8 tests. The results of these calculations are presented in Table 5, which includes the means and SDs for each of the eight BrainCheckers tests for the two subject groups as well as the group and error sum of squares, and the *F* statistics with their associated probabilities. The calculations summarized in Table 5 demonstrated that at MSL1 there is no evidence of any difference in the performance between the two groups of subjects on any of the tests.

**Table 5:** **MSL1 means and SDs of the eight BrainCheckers test scores for the mTBI and the control subjects along with the results of the ANOVA**.

Measure	Group	Mean	SD	Sum of square		*F*(1, 70)	Probability
				Group	Error	
Sleep scale							
Rating	mTBI	1.39	0.688	0.681	22.194	2.146	0.147
	Control	1.19	0.401	
Simple reaction time							
Throughput	mTBI	197.39	27.93	6.722	56006	0.008	0.927
	Control	196.78	28.63	
Procedural reaction time							
Throughput	mTBI	105.47	13.67	213.556	9155	1.633	0.206
	Control	108.92	8.65	
Running memory continuous performance							
Throughput	mTBI	88.25	23.84	0.889	38587	0.002	0.968
	Control	88.03	23.11	
Matching to sample							
Throughput	mTBI	37.14	12.29	1.389	9645	0.01	0.92
	Control	37.42	11.16	
Congruent stroop							
Throughput	mTBI	88.08	16.1	8.681	13805	0.044	0.834
	Control	87.39	11.64	
Incongruent stroop							
Throughput	mTBI	73.58	15.95	91.125	21532	0.296	0.588
	Control	71.33	18.99	
Pursuit tracking							
% Time on target	mTBI	88.25	8.34	5.556	6132	0.063	0.802
	Control	87.69	10.28	

### Difference scores

Predicated on the results of the comparisons summarized in Table 5, difference scores were calculated, as described above, for each of the BrainChecker tests for the 8, 12, and 14k altitude conditions. Since the three different altitude conditions are confounded with order effects they were combined into a single experimental treatment. Table 6 shows the means over the three altitude conditions for each of the BrainChecker tests for each group of subjects. Table 6 also includes the Standard Error of the Means (SEM) and the results of the between-group ANOVA.

**Table 6:** **Difference scores for each of the BrainChecker tests for the two groups of subjects and the results of the between-group ANOVA**.

Test	Group	Mean	SEM	Sum of squares		*F*(1, 70)	*p*
				Group	Error	
Sleep rating	mTBI	−0.481	0.103	0.116	79.88	0.101	<0.751
	Control	−0.528	
Reaction time	mTBI	−3.37	3.724	0.167	104817	0	<0.992
	Control	−3.315	
Procedural reaction time	mTBI	4.167	1.268	10.277	12148	0.059	<0.809
	Control	3.731	
Running memory	mTBI	−8.222	2.627	148.338	52164	0.199	<0.657
	Control	−9.88	
Matching to sample	mTBI	6.333	1.102	766.894	9180.546	5.847	<0.018
	Control	2.565	
Congruent stroop	mTBI	−3.463	1.468	130.667	16297	0.561	<0.456
	Control	−5.019	
Incongruent stroop	mTBI	−3.213	1.539	4.741	17907	0.019	<0.892
	Control	−2.917	
Pursuit tracking	mTBI	1.981	0.998	5.352	7524.63	0.05	<0.824
	Control	1.667		

Of the BrainCheckers tests, M2S was the only test in which the performance of the two subject groups under hypoxic stress was statistically different [SS Group = 766.894, SS Error = 9180.546, *F*(1, 70) = 5.847, *p* < 0.018]. Specifically, mean throughput difference for the mTBI group was 6.3331 whereas the mean throughput difference for the controls was 2.565. It should be noted that the way the throughput difference is calculated, the greater the number, the bigger the performance degradation associated with altitude.

The difference between the mTBI (mean = 4.64, SD = 9.424) and the control subjects (3.17, SD = 8.997) M2S MSL2 throughput difference scores was not statistically different [SS Group = 39.014, SS Error = 5941.306, *F*(1, 70) = 0.460, *p* > 0.50], indicating that whatever effects the hypoxic exposure had, the effects were not evident during the MSL2.

For the sake of completion, Table 6 includes the means, the SEM, as well as the ANOVA results for the BrainCheckers tests that were not significant.

## Discussion

The present data show that under low stress conditions, the performance on the M2S test, a measure of short-term visual memory, of persons with mTBI was comparable to that of healthy controls; but when stressed, persons with mTBI showed a disproportionately severe impairment on this test. Thus, persons with mTBI may appear normal when examined under conditions of low stress, such as are typical during routine clinical examinations, but may exhibit worrisome memory dysfunction when exposed to relatively mild and common stressors. This may be an operationally important finding relevant to operators who obtain information by rapidly scanning a visual display and interpreting differences in the display pattern over time, as is the case with flight instrument displays. This idea may help explain the effect of hypoxia on the one pilot’s flight performance described above.

Our findings suggest that the stressor used in the present study, hypoxia, has the potential of being the basis for a practical “brain stress test” analogous to the standard cardiac stress test. Such a capability would be particularly important since mTBI, even when apparently completely recovered using conventional examination strategies, may include deficits observable only under stress.

Seven of the BrainCheckers tests showed no difference in the effects of the hypoxic stress on the two groups of subjects, suggesting that the effect of the stress on cognitive function was relatively specific and not a generalized decrement in cognitive function. The hypoxia literature, while inconsistent in terms of identifying specific cognitive functions most vulnerable to brief hypoxic exposure, raises the question of why a greater number of the BrainCheckers subtests failed to show an effect (Petrassi et al., 2012). This may be explained by the very mild nature of the concussed group’s symptoms. Recall that at MSL1 and MSL2 both groups showed virtually identical cognitive performance, suggesting that cognitive abnormalities in the concussed group, if present, would be very mild and subtle. It is possible that M2S is more sensitive to hypoxia-induced cognitive impairment, or even to the subtle, lingering remnants of distant mild concussions. This also raises the possibility that different cognitive functions may be differentially vulnerable to different physiological stressors, for example, alcohol or drug effects may impede cognition in ways that are different than normobaric hypoxia or sleep deprivation.

While this study is consistent with the study by Ewing et al. (1980), it suffers from several additional limitations. One is the use of subjects’ self-report of their medical and concussion histories. Another is the wide range in the interval between the subjects’ time of injury and their time of study participation. Moreover, the study examined cognitive performance only on a brief battery of computerized cognitive tasks, but did not include comprehensive neuropsychological assessment. These weaknesses emphasize the importance of replication and further study.

A possible explanation for the latent deficits exposed by hypoxic stress is that mTBI patients have to “work harder” in order to perform normally, so they are closer to their maximum capabilities and more vulnerable to resource depletion when stressed. Recent studies showing fMRI and EEG abnormalities in patients with normal neuropsychological testing support this possibility (Gosselin et al., 2006; Broglio et al., 2009; Pontifex et al., 2009; Slobounov et al., 2010).

Given the heterogeneity of stressors to which Warriors routinely are exposed, the effects of concussion upon resilience to such stressors urgently require further study. The stress in the current study, hypoxia, may be a unique stressor for unmasking latent memory impairment following concussion, or, as further research may show, other stressors also may have disproportionately negative effects upon diverse cognitive functions.

The “hypoxic challenge” paradigm should be further explored for its potential clinical utility. In particular, future studies should include assessment of subjects’ ability to discern whether they are at MSL or under hypoxic challenge. If subjects cannot discern between the two conditions, but the present findings are replicated, the hypoxic challenge paradigm may be less vulnerable to effects of dissimulation for secondary gain or diminished effort secondary to depression and other conditions that frequently are co-morbid with mTBI. If this is the case, hypoxic challenge would prove an extremely valuable assessment tool for mTBI patients with latent deficits that current neuropsychological assessments do not detect.

In support of this potential utility it should be noted that none of the subjects reported any difficulties with the hypoxic stress used in the present study. Furthermore, the subjects set their own pace for completing the experiment including the duration between the three successively severe normobaric hypoxic stress conditions. There was no evidence that the mTBI subjects needed longer breaks between exposures than did the controls, supporting the notion that both subject groups sustained the stress equally well.

## Conflict of Interest Statement

The authors declare that the research was conducted in the absence of any commercial or financial relationships that could be construed as a potential conflict of interest.
